# Patient Experiences of Nutrition in Enhanced Recovery After Colorectal Surgery: A Systematic Review †

**DOI:** 10.3390/nu18111790

**Published:** 2026-06-01

**Authors:** Kimberly Yee Hooi Ang, Georgia Stringer, Jorja Collins, Lisa A. Barker

**Affiliations:** 1 Department of Nutrition and Dietetics, Box Hill Hospital, Eastern Health, Box Hill, VIC 3128, Australia; 2 Department of Nutrition, Dietetics and Food, Monash University, Notting Hill, VIC 3168, Australia

**Keywords:** enhanced recovery after surgery, colorectal surgery, patient experiences, qualitative, nutrition, systematic review

## Abstract

**Background and Objectives:** Perioperative nutrition is a core component of Enhanced Recovery After Surgery (ERAS) pathways. Understanding the patient experience of nutrition recommendations provides insight into the acceptability of perioperative nutrition care and facilitates the achievement of ERAS targets. This systematic review aimed to synthesise patients’ experiences of nutrition within ERAS pathways for colorectal surgery. **Methods:** A systematic search of Ovid MEDLINE, Embase, Emcare, and CINAHL was conducted to identify studies published up until July 2025. Eligible studies included qualitative, mixed-methods, or descriptive survey designs. Data were extracted and synthesised using an inductive thematic analysis. Methodological quality was assessed using the Mixed Methods Appraisal Tool. **Results:** Fifteen studies were included (40% qualitative, 33% quantitative, 27% mixed-methods), representing data from 1431 patients. Eleven studies met all quality criteria. Five themes were identified. Information gaps and misconceptions about nutrition (Theme 1) resulted from unclear advice across care settings. Oral intake post-surgery (Theme 2) was limited by nausea, reduced appetite, early satiety, and dissatisfaction with hospital food. Experiences with oral nutritional supplements (Theme 3) were variable, with palatability affecting acceptability. Healthcare professionals (Theme 4) were central in shaping patient confidence in nutrition care. The transition to home (Theme 5) was a vulnerable period where follow-up support was highly valued. Heterogeneous reporting of nutrition in ERAS contexts was a limitation. **Conclusions:** Patient engagement with ERAS nutrition is shaped by individual and healthcare system factors. Addressing information gaps, providing nutrition support, and integrating patient perspectives through codesigned education and research initiatives may enhance perioperative nutrition experiences and optimise recovery outcomes.

## 1. Introduction

Traditional post-operative recovery protocols following colorectal surgery commonly emphasise gut rest, resulting in delayed oral intake, postponed mobilisation, and prolonged hospitalisation. Historically, patients were often restricted from eating or drinking until bowel function had resumed, based on a belief that this approach protected gastrointestinal integrity. However, this practice was frequently associated with adverse outcomes, including physical deconditioning, delayed wound healing, increased risk of malnutrition and increased postoperative complications. Emerging evidence has since challenged this paradigm, demonstrating that early postoperative feeding can support recovery and improve clinical outcomes [1]. In recent decades, the introduction and growing implementation of Enhanced Recovery After Surgery (ERAS) programmes internationally have transformed perioperative care by promoting a multimodal, multidisciplinary, and evidence-informed approach to surgical recovery [2].

ERAS is an optimised perioperative care pathway designed to attenuate surgical stress responses [3]. As a result, ERAS pathways have been shown to reduce complications and expedite post-operative recovery. It has been successfully adopted in multiple surgical disciplines, including colorectal, gynaecological, orthopaedic and other surgical settings [4]. ERAS in practice can also be referred to as an Enhanced Recovery (ER) pathway.

Perioperative nutrition is a fundamental component of ERAS pathways. Nutrition optimisation strategies can promote recovery for surgical patients who are at increased risk of malnutrition and gastrointestinal dysfunction. Core nutrition-related elements include preoperative carbohydrate loading, avoidance of prolonged fasting, early resumption of oral intake, and the use of oral nutritional supplements (ONS) [1]. The clinical benefits of perioperative nutrition within ERAS protocols are well established. High-quality evidence demonstrates that ERAS pathways are associated with improved surgical outcomes [2,5]. Systematic reviews and international guidelines consistently identify perioperative nutrition as a core component of these pathways, with benefits largely demonstrated as part of the ERAS bundle rather than as isolated interventions. Specifically, in a 2025 systematic review, compared with conventional care, ERAS patients had improved clinical markers of nutritional recovery [6].

Perioperative nutrition is particularly relevant for colorectal surgery, where targeted ERAS nutrition strategies directly influence postoperative outcomes [7,8]. In colorectal surgery, these nutrition-related strategies are incorporated into ERAS protocols to support gastrointestinal recovery and reduce postoperative complications [4]. The ERAS Society guidelines for perioperative care in colorectal surgery also outline perioperative nutrition as having a strong grade of recommendation [7]. Similarly, the European Society for Clinical Nutrition and Metabolism (ESPEN) Practical Guideline for Surgery recommends high compliance with nutrition elements of metabolic control and avoiding long preoperative fasting [9]. Perioperative nutrition in ERAS protocols has distinct benefits in improving colorectal surgical outcomes, such as reduced postoperative insulin resistance, lower infection rates, improved anastomotic healing, decreased length of hospital stay, and faster return of bowel function [2,10].

Despite this strong clinical basis for the role of perioperative nutrition within ERAS, little is known about the real-world patient experience. Patients’ understanding, beliefs, and expectations substantially influence their engagement with perioperative nutrition recommendations. It is also important to consider how socio-ecological factors, healthcare systems and structural constraints can shape patient experiences and behaviours. For example, it is vital to understand how patients interpret nutrition advice and manage their nutrition-related symptoms within the constraints of the healthcare system. Factors such as access to nutrition information, symptom management, and education may be vital to patient compliance and improved outcomes.

Exploring patients’ experiences of nutrition within ERAS pathways provides insight into factors that shape their acceptability and adherence to perioperative nutrition care and, in turn, influence whether the clinical benefits of ERAS are achieved. To date, no systematic review addressing patient experiences of nutrition within colorectal ERAS pathways has been completed. This systematic review aims to synthesise the perceptions, experiences, attitudes, knowledge, and views on the role of nutrition by patients undergoing colorectal surgery within ERAS pathways.

## 2. Methods

This systematic review followed the framework set out by Preferred Reporting Items for Systematic Reviews (PRISMA) [11]. The full PRISMA checklist can be found in Table S1 of the Supplementary Material. The protocol was published in PROSPERO prior to review preparation (CRD420251005025).

### 2.1. Eligibility Criteria

The Population, Intervention, Comparator, and Outcome (PICO) framework, first proposed by Richardson et al., was used to formulate the inclusion and exclusion criteria [12]. The inclusion criteria are highlighted in Table 1. No date or language limits were applied.

### 2.2. Information Sources

A systematic search of the electronic databases Ovid MEDLine, Embase (Ovid), Emcare (Ovid) and CINAHL (EBSCO) was completed in September 2024 and re-run in July 2025, including all articles published until the search date to ensure the search was current.

### 2.3. Search Strategy

The search strategy was developed by KA and discussed with JC and LB. This was refined under the consultation of a specialist librarian to support the identification of appropriate keywords and synonyms to optimise retrieval across databases. Searches were also adapted for individual databases in consultation with the librarian.

Search terms were systematically developed and organised into three key conceptual domains to comprehensively capture the relevant literature: (1) ERAS, (2) patient experience, and (3) colorectal surgery. This structured approach ensured that the search strategy aligned with the review aim and identified studies exploring patients’ perceptions of nutrition within colorectal ERAS pathways. For each domain, a combination of free text keywords and controlled vocabulary terms, such as Medical Subject Headings [MeSH] and equivalent thesaurus terms, was used where applicable to maximise search sensitivity and account for variations in terminology across databases. The search terms categorised in the three conceptual domains are outlined in Tables S2 and S3 of the Supplementary Material. The full search strategies for all databases, including keywords, controlled vocabulary and Boolean operators, are outlined in accordance with PRISMA recommendations. The search strings are listed in Table S4 and File S1 of the Supplementary Material.

### 2.4. Selection Process

Searches were imported into Covidence (Covidence systematic review software, Veritas Health Innovation, Melbourne, Australia), an online systematic review management platform. This software facilitated the selection of appropriate studies and removed duplicates. Titles and abstracts were screened independently in duplicate by two reviewers (KA and either JC or LB). KA, JC and LB performed full-text reviews. At all steps of title, abstract and full text assessment, discrepancies were resolved through discussion between reviewers, with consensus reached among KA, JC and LB.

### 2.5. Data Collection Process

Data were extracted independently in duplicate by KA and either GS or LB, with reviewers blinded to each other’s extraction. Following extraction, data were compared, and discrepancies were resolved through discussion and consensus among KA, GS and LB. A data extraction form in Microsoft Excel (Version 16.109.2, Microsoft Corporation, Redmond, WA, USA) developed by all authors was used for data extraction. Where a study reported both broad ERAS experiences and nutrition-related results, only the nutrition-relevant text and results were extracted. Nutrition-specific findings were prioritised when they directly addressed the review question, reflected patient experiences of a nutrition element of ERAS, or helped explain barriers or facilitators to engagement with perioperative nutrition. 

### 2.6. Data Items

Data extracted from each study included: title, author and year of publication; country and healthcare setting; study aim and study design; funding sources; participant characteristics (including sample size and if they were patients or caregivers); timing within the perioperative pathway; description of local ERAS pathway; nutrition-related extractable findings; data collection methods; key findings related to patient perceptions, experiences, attitudes, knowledge, or views of nutrition; implications for practice; strengths and limitation of each study; and reported barriers and facilitators to engagement with perioperative nutrition.

### 2.7. Risk of Bias Assessment

Study quality was assessed using the Mixed Methods Appraisal Tool (MMAT 2018) [13]. The MMAT comprises two initial screening questions to determine the eligibility of studies for appraisal. Papers are then categorised according to their study design of either qualitative, quantitative, randomised controlled trials, quantitative randomised, quantitative descriptive, or mixed methods. A subsequent five questions under each study design type are used to assess methodological rigour and relevance. Each section, based on study design, has specific criteria, with studies rated as “Yes”, “No”, or “Can’t Tell” based on the degree to which these items are satisfied.

MMAT does not provide a single overall score but facilitates an understanding of study quality by focusing on strengths and weaknesses in specific domains. The MMAT discourages excluding studies with low methodological quality. Instead, there is the potential to consider the quality of the studies by comparing the contrast in results. Two authors independently assessed and rated the quality of all studies in duplicate (KA, with either GS or LB). Any disagreements or incongruities were discussed between KA, GS and LB.

### 2.8. Synthesis Methods

A narrative synthesis of data from included studies was undertaken. Extracted data comprised study characteristics and outcome data, including both qualitative findings (such as interview and focus group data) and quantitative descriptive findings (such as survey responses related to patient experiences). Quantitative findings were treated as descriptive outcome data and integrated alongside qualitative findings where they addressed patient perceptions, experiences or attitudes towards perioperative nutrition. Outcome data were analysed using a thematic synthesis approach as described by Braun and Clarke [14]. This was undertaken by KA, who is a clinical dietitian with 6 years of experience, including 3 years of experience in colorectal surgery. Extracted findings were coded inductively, with initial codes generated from participants’ reported experiences and interpretations. Codes and patterns of codes across studies were iteratively reviewed, grouped, and refined into themes that captured patients’ perceptions and experiences of nutrition care in the context of ERAS. To enhance reliability, coding and theme development were reviewed and discussed throughout the process with GS and LB. The findings were coded by KA and independently double-coded by GS or LB. The final theme map was consolidated with all three reviewers to ensure alignment among the coding. Themes were refined through iterative discussion to ensure they accurately reflected the underlying data and were consistent across studies.

## 3. Results

### 3.1. Study Selection

**Figure 1:**
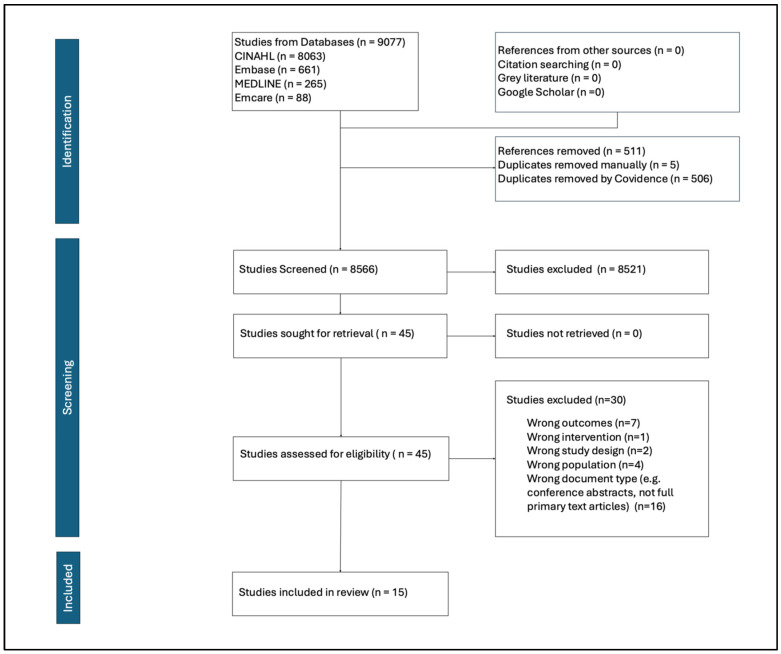
PRISMA Flow diagram of study identification, screening and inclusion.

The initial database searches completed on 10 September 2024 identified 8469 records after removal of duplicates. Figure 1 provides further detail of study selection. The literature search was re-run on 16 July 2025, identifying a further 97 records, with two additional studies published in 2024 subsequently included. In total, 8566 records were screened, 45 articles were assessed in full, and 15 studies were included in the final synthesis [14,15,16,17,18,19,20,21,22,23,24,25,26,27,28] (Figure 1).

### 3.2. Study Characteristics

Table 2 summarises the characteristics of the 15 included studies, representing data from 1431 patients. Six studies (40%) used qualitative methods, 5 studies (33%) used quantitative survey-based methods, and 4 studies (27%) used mixed methods. Sample sizes varied considerably. Smaller samples were more typical of qualitative studies (ranging from 10 to 27 participants), whereas larger samples were predominantly observed in surveys, including two large studies with 286 and 652 participants, respectively. All included studies exclusively reflected patient perspectives. Although caregivers were included within the eligibility criteria, no studies specifically examining experiences were identified in the available literature. 

The studies were conducted across a range of countries, most frequently in the United Kingdom (*n* = 6), followed by Canada (*n* = 3), Sweden (*n* = 3), and single studies from Belgium, Spain, China, and France. All studies were published in English, although several were conducted in non-English-speaking contexts. Researchers were also of varied professional backgrounds, including surgeons, nurses, clinical researchers and dietitians.

### 3.3. Risk of Bias Assessment Results

All quality criteria were achieved in six out of six qualitative studies, indicating consistently high methodological quality within this study design. In comparison, all quality criteria were met in 4 out of 5 quantitative descriptive studies and 1 out of 4 mixed methods studies. Mixed-methods studies showed the greatest variability in quality and the lowest quality, with limitations most commonly related to the integration of qualitative and quantitative components and justification of mixed-methods design. Table 3 displays the full results of the methodological quality appraisal. Mixed-methods studies were appraised using the MMAT mixed-methods criteria (MM1–MM5), whereas quantitative descriptive studies were appraised using QD1–QD5.

### 3.4. Outcomes

There were five themes identified describing patient experiences related to nutrition in ERAS in colorectal surgery. These themes highlight barriers, knowledge gaps and the understanding of the role of nutrition in the perioperative journey. These themes are:

1. Information Gaps and Misconceptions About Nutrition
2. Factors Impacting Oral Intake Post-Surgery
3. Experiences with Oral Nutritional Supplements (ONS)
4. Role of Healthcare Professionals
5. Transition to Home and Discharge Care

Table S5 in the Supplementary Material presents a summary of findings contributing to each theme.

Themes were differentiated according to their primary analytic focus during coding and synthesis. Theme 1 captured patient knowledge, beliefs, and misconceptions about perioperative nutrition. Theme 2 focused on environmental and physiological influences on oral intake after surgery. Theme 3 addressed the acceptability and use of oral nutritional supplements. Theme 4 looked at the influence of healthcare professionals and their delivery of nutrition communication. Theme 5 addressed experiences of nutrition care during discharge. Although some findings overlapped across themes, codes were iteratively compared across studies and assigned to the theme that best reflected the central meaning of the patient experience described.

**Theme 1: Information gaps and misconceptions about nutrition**

The first theme was identified in eight of the included studies [16,20,22,23,26,27,28,29]. These studies described patient-perceived gaps in the understanding of perioperative nutrition. Across studies, findings showed uncertainty about the purpose of early feeding and therapeutic diets.

This contributed to hesitation or reduced adherence to ERAS nutrition protocols. For example, in Gillis et al. (2017), patients described how a lack of understanding of the rationale for the ERAS protocol affected their engagement with ERAS nutrition elements [22].

“I did it, but didn’t know why. I think people would be more diligent if they knew why the walking was so important, why the protein was so important…”

[22]

Patients who received clear and meaningful explanations appeared more willing to engage with nutrition recommendations. Gillis et al. (2019), found that when patients received structured nutrition education as part of their ERAS journey, they tended to believe in the healing value of nutrition in optimising their surgical outcomes [23]. Similarly, Taylor and Burch (2011) found that food provoked strong emotions during the ERAS pathway, and patients understood the importance of eating [27].

Information gaps were not just a matter of patients lacking facts, but reflected a broader mismatch between ERAS nutrition recommendations and patients’ pre-existing beliefs about eating after bowel surgery. There was a misconception regarding the need for postoperative “bowel rest”, leading some patients to intentionally delay oral intake despite ERAS guidance. Gillis et al. (2019) highlighted this as one of their main findings contributing to poor adherence to early oral feeding, explained by the quote from a patient:

“vaguely remember them bringing me a full tray at suppertime…are you guys nuts? I didn’t touch it because I felt let’s not overtax the bowel.”

[23]

This theme is reflected by Wennstrom et al., with a patient demonstrating a fear of bowel dysfunction with early oral feeding [28].

“The first meal consisted of ropy meat, pineapple pie and long pieces of vegetables (peppers) with the skin left on, which is exactly what you should avoid when you have just had surgery and there’s a risk of slowed-down bowel movements.”

[28]

Some studies demonstrated that patients rely on prior experiences or personal interpretations of what was safe to eat. A satisfaction survey by Olivares et al. showed 58.2% of patients felt it was either too early or somewhat early to commence oral fluids in the first 24 h postoperatively, despite most (63.2%) of patients not experiencing any nausea or vomiting postoperatively [20]. Similarly, in the nurse-led follow-up study by Burch and Taylor (2012), it was reported that patients deliberately disregarded professional dietary advice because they feared that eating could harm bowel function [19]. Short et al. found that some participants wanted to rest the digestive system [26]. They also found that this relationship was more complicated when a stoma was formed [26]. Patients perceived stomas as an extreme bodily change, resulting in a substantial shift in their perception of food. Patients felt unclear as to which foods would be ideal to accommodate a stoma and were wary of foods that would increase output or cause blockages [26]. One participant even reduced food intake in an effort to control their stoma output:

“Everything that goes in my mouth I can see coming out and that’s quite off-putting… I think subconsciously I’m probably holding back more on what I’m eating.”

[26]

Patients also misunderstood the purpose of prescribed postoperative diets, particularly low-fibre and high-energy and high-protein regimens, which were perceived as unhealthy rather than therapeutic measures to support bowel recovery. Gillis et al. (2017) reported patients wanting clearer guidance about food choices, with those who had been warned about the postoperative low-fibre diet being “more forgiving” [22]. This highlighted how inadequate explanations contributed to negative perceptions of these diets.

When there were fewer information gaps and a greater understanding of ERAS benefits, patients were more willing to participate in ERAS protocols. In the questionnaire by Xu et al., a positive correlation was found between greater ERAS understanding and better participation in ERAS practices [29]. Similarly, S Ben Ali et al. found that higher patient activation, defined as having knowledge, skills and confidence for patients to participate in their care, was associated with better adherence to ERAS protocols [16]. Overall, these studies suggest that engagement with ERAS nutrition is shaped by information provided and by whether that information is made meaningful within patients’ own understanding of their recovery.

**Theme 2: Factors impacting oral intake post-surgery**

The second theme was split into subthemes of physiological factors related to eating and interactions with the hospital foodservice environment. In the included literature, postoperative oral intake was shaped by a complex interaction between patients’ physiological symptoms and the hospital food environment. Across studies, barriers to postoperative eating included the management of physiological symptoms within the hospital food environment. Four studies reported that nausea, vomiting, early satiety, fatigue, pain and altered bowel function limited patients’ ability and willingness to eat during early recovery [24,25,26,28]. These studies also reported that some patients delayed oral intake in response to these symptoms. In the Swedish ERAS cohort reported by Wennstrom et al., more than half (55%) of participants reported reduced or absent appetite following discharge [28]. In the study by Short et al., fear of gastrointestinal discomfort, nausea and vomiting was reported [26]. Some individuals reported distress at the idea of vomiting, and felt it was normal to have a reduced appetite in the hospital [26]. Although patients were generally aware that early oral intake was encouraged within ERAS, symptom burden and fear of recurrence acted as powerful deterrents. Another participant in the same study articulated how their perception of having surgery shaped eating behaviour:

“when I’ve been sick, I feel I want to give my stomach a rest… once I’ve been sick I will tend to say… Well, I won’t have any more food now for a while.”

[26]

In the survey by Partoune et al., approximately one-third of patients described difficulty eating due to loss of appetite and early satiety [24]. Several patients suggested that a slower diet upgrade would have been more tolerable for them [24]. Similarly, Samuelsson et al. found patients had difficulties recovering from surgery due to fatigue, pain and bowel dysfunction [25]. Patients were disappointed in the lack of effort to tailor nutrition to their individual situations [25]. Reduced oral intake after surgery reflects the interplay between patients’ physiological symptom burden and their interpretation of these symptoms.

Patient experiences with hospital foodservice environments were identified in eight studies [18,21,22,23,25,26,27,28]. In some studies, patients reported hospital food not meeting their perceived quality standards. Samuelsson et al. found that all participants complained about hospital food quality, expressing “astonishment that such a vital part of recovery, that is gaining weight and thereby strength, is neglected” [25]. In the questionnaire follow-up study by Burch (2015), one participant stated, “Something seriously needs doing about the food” [18]. The study highlighted that hospital food was the most frequent comment when asked about how to improve their hospital stay [18].

Taylor and Burch (2011) highlighted that while participants understood the importance of eating as part of the ERAS protocol, unappetising hospital food was one of the factors hindering engagement with eating [27]. One participant stated:

“I got to the stage that had I stayed for another evening meal I would have walked down to the canteen to get some decent food.”

[27]

Oral intake appeared to be shaped by the extent to which symptoms were addressed through responsive care practices and whether the food environment supported eating as a component of recovery, rather than positioning it as an added burden. Gillis et al. (2019) found that complaints about the hospital food were common, attributing these to personal food preferences [23]. These findings acknowledged that the hospital foodservice system was not tailored specifically to patient preferences, and patients enjoyed food much more when it was brought from home [23]. Gillis et al., in their 2017 paper, also found interruptions to mealtimes, missed meals, and inability to open the food trays were barriers to adequate oral intake [22].

Only one paper, a questionnaire of 105 patients participating in an Enhanced Recovery pathway by Cooper, found positive perceptions about the hospital food [21]. It was found that there was a high degree of variation in how patients perceived the hospital food; however, 76% responded that it was “average” or “good” [21]. This paper did not report on the impact of oral intake that this perception resulted in.

Wennstrom et al. found that hospital dining environments could help patients to engage in eating [28]. One patient described, “I felt fine the whole time sat up walked to the dining room ate and socialized with other patients.” [28] A shared dining room environment was highlighted as a facilitator of helping patients in similar situations socialise and share experiences [28].

**Theme 3: Experiences with oral nutritional supplements**

Three studies explored patients’ experiences of ONS as part of ERAS pathways [17,26,27]. Across these studies, patients acknowledged the role of ONS in supporting recovery, yet constantly reported difficulties adhering to them in practice. A clear pattern emerged in which acceptability was largely determined by the sensory experience of consumption, with all three studies identifying palatability as a major challenge. Brundrett et al. found that participants recognised that supplements were intended to support recovery [17]. However, patients often disliked the sweetness, heaviness, or texture of ONS, which impacted their enjoyment of the ONS and how easily they were able to consume the drink [17]. In the focus group study by Taylor and Burch (2011), participants described aversions to the taste and texture of ONS, yet felt compelled to consume them because they believed they were beneficial for healing:

“The energy drinks were horrible and yet you felt you ought to try to get them down, as I was told they were good for body repair.”

[27]

These findings indicate that tolerance of ONS is shaped by the interaction between sensory perceptions, postoperative symptom burden, and patients’ understanding of the purpose of ONS in their recovery, all of which influence adherence.

Short et al. found that patients were confused about the differences between preoperative carbohydrate loading drinks and postoperative ONS [26]. Furthermore, patients were less willing to consume the postoperative nutrition drinks compared to the preoperative carbohydrate drinks [26]:

“It was, “What do you want for a drink?” and the first couple of days I said “yes” and then I thought “I don’t really enjoy these, they are just so sweet…”

[26]

This suggests that the perceived benefit of ONS did not consistently translate into ongoing compliance.

**Theme 4: The role of healthcare professionals**

Seven studies highlighted the central role of healthcare professionals in shaping patients’ engagement with perioperative nutrition [15,18,22,23,25,26,29]. In these studies, patients described how clinician interactions were pivotal in influencing their understanding and adherence to ERAS nutrition recommendations. The lack of such resources was perceived negatively. Healthcare staff interactions during their hospital stay were described and seen as key in reinforcing nutrition recommendations, providing day-to-day encouragement for oral intake and acting as primary points of contact for patient questions. A consistent pattern across studies was that patients felt more confident engaging with ERAS when communication was clear, timely and responsive. In contrast, interactions that were rushed, inconsistent, or impersonal weakened trust and diminished participation in ERAS nutrition protocols.

Gillis et al. (2019) identified that the role of the dietitian was particularly important [23]. The patients who met with a dietitian felt they offered helpful nutrition recommendations and compassion, suggesting patients valued high-quality and specific nutrition information.

“I think the best information that I retained and took hold was on nutrition. That was really, really good.”

[23]

Similarly, Aasa et al. reported that a preoperative face-to-face consultation with a nurse enhanced patients’ sense of preparedness and security in proceeding with surgery [15]. This interaction strengthened their willingness to mobilise, eat and take ownership of their recovery [15].

“I felt taken care of in a good way, they care about what happens to you, it felt good.”

[15]

Across studies, clarity and responsiveness in communication from healthcare professionals emerged as a pivotal determinant in patient engagement. Adherence was supported most effectively when clinicians translated ERAS nutrition principles into personalised, meaningful guidance rather than delivering generic protocol instructions.

However, healthcare professionals could also become a source of uncertainty. Several studies noted that patients desired more consistent and reliable nutrition advice. When this was absent, they perceived a gap in care and expressed dissatisfaction [21,25,28]. Short et al. found this, and also found that patients viewed the internet as an unreliable source [26].

“I’ve asked both the oncologist and the surgeon whether I should or should not be having or following a particular diet or avoid eating certain things and there was no specific information…”

[26]

Similarly, Gillis et al. (2017) found that patients sought more nutrition information, seeing the lack thereof as a missed opportunity, with some conducting their own internet research [22]. In the survey of 702 patients conducted by Xu et al., it was found that 75.07% of patients felt receiving nutritional support before surgery would be helpful [28]. However, the paper does not define ‘nutritional support’, whether it refers to a dietitian consultation or to other nutrition support measures such as ONS.

The interactions with healthcare staff in the hospital shaped patient perceptions of their recovery journey. Samuelsson et al. described patients perceiving medical staff as busy and stressed. Therefore, communication was perceived as hasty, and information was difficult to understand. This caused patients to feel a loss of control and anxiety, which affected their engagement in their recovery [25].

“You can see that the staff has a lot to do, so you feel reticent even though you need to ask a question.”

[24]

Similarly, Burch (2015) found good feedback about the professionalism of staff and nursing care; however, the author highlighted patients’ desire for more nursing staff during the inpatient stay [18]. Gillis et al. (2017) described rapport with nursing staff as variable, with some nurses being very informative and others being less interested [22]. Being well-engaged with the healthcare staff helped patients understand the ERAS programme, which helped adherence [22]. Conversely, some staff were perceived as being fixated on following the ERAS protocols and not accounting for individual conditions [22]. One participant reported anxiety around this:

“The nurses and doctors were pushing me to eat. I did not understand how important it was to eat as soon as possible. I thought the body needs healing…”

[22]

The same paper highlighted that receiving information that was reiterated by multiple healthcare professionals helped patients feel more secure in what to do after surgery.

**Theme 5: Transition to home and discharge care**

The fifth theme was found in five studies [19,22,25,26,27,28]. Discharging home was seen as an avenue of opportunity to progress eating. Yet, it was also viewed as a vulnerable period marked by uncertainty, reduced confidence and inadequate ongoing support. Across studies, patients anticipated that returning home would make eating easier, as being in their own environment offered familiar foods and a return to normal routines. However, the transition home exposed uncertainties that had been less apparent in the hospital. Patients reported new concerns about what to eat and where to seek help if recovery did not progress as expected.

For some patients, being discharged home was perceived as an enabler to progress their oral intake. Short et al. found that despite patients experiencing physiological challenges such as loss of appetite, nausea and vomiting, patients were strongly motivated to progress their eating when faced with the prospect of returning home [26]. Patients had the perception that returning home would restore a sense of normality in their routine, thus enabling oral intake.

“I’m gonna try a bit again today… obviously I’ve got to eat my food before I can go home… if I don’t eat then I won’t be allowed to go home…”

[26]

Similarly, Wenstrom et al. found 80% of patients felt more comfortable when coming home because they had access to selecting their own food [28].

However, for many patients, symptom burden and uncertainty in knowing what they would tolerate eating or were allowed to eat was a barrier to participating in eating. Short et al. found that patients held doubts about reduced activity and the diet changes required when returning home [26].

“I was told…when I go home and I’m able to eat again, I can eat or drink anything I want… hopefully I’ll go back to how I was eating and drinking before.”

[26]

Burch and Taylor (2012) also found that 29% of patients perceived they were consuming half or less of their usual diet after discharge, with contributing factors of concerns with bowel function and fatigue [19]. These findings suggest that the transition from a structured hospital environment to a home setting made the responsibility for managing recovery more tangible and, for some patients, more challenging.

The presence of follow-up support was perceived by patients as an important factor in providing clarity around oral intake. A lack of follow-up support caused feelings of vulnerability and anxiety around eating. Gillis et al. (2017) found that patients experienced confusion and anxiety when there was a lack of information given on their post-discharge diet [22].

“The take home sheet that I had, it did say eat more small meals and make sure you drink lots of water. Helpful hints. I wanted more detail like that.”

[22]

Similarly, Samuelsson et al. reported that many patients experienced nutritional problems and weight loss [25]. There was also confusion regarding the best point of contact for questions, causing feelings of abandonment and reduced safety.

“I called the contact nurse who said that I should ring the primary care centre, but they said that the operation was the hospital’s responsibility. Then they said: let’s not concern about this anymore; from now on you can fix this yourself.”

[25]

When there was postoperative support provided by a healthcare professional, it was highly valued by patients. It was noted that few were contacted by a dietitian post-discharge. However, when patients did receive dietitian support, it provided patients with assurance that their nutritional status was being monitored and created a sense of safety [25]. Similarly, Wenstrom et al. found that all patients appreciated a nurse calling them one week postoperatively, providing a sense of safety and assurance [28]. Patients appeared to manage the transition home more confidently when discharge nutrition advice was specific and clear, and available follow-up avenues existed.

## 4. Discussion

This systematic review synthesised literature describing patients’ experience of nutrition in colorectal ERAS protocols, which adds patient perspectives to the body of ERAS literature that is currently dominated by clinical and health service outcomes. The results from this review demonstrate that patients’ access to information and support from health professionals positively impacts their understanding and confidence in postoperative recovery. Physiological barriers, hospital foodservice factors and the palatability of oral nutrition supplements influenced patients’ interactions with oral intake. These findings highlight that engagement with ERAS nutrition is shaped not only by patients’ individual understanding and experiences of ERAS pathways but also by broader systemic and contextual factors.

Patients frequently reported uncertainty regarding pre- and postoperative nutrition expectations, leading to confusion, anxiety, and misconceptions about appropriate dietary practices. Unlike other ERAS components, eating is a familiar and highly personal aspect of care. In the absence of clear, condition-specific guidance, patients often rely on prior experiences and cultural beliefs to guide food choices [30,31]. Dietary choice has also been linked to several external influences, including varying dietary advice from healthcare professionals, self-sought out online sources or nutrition advice from friends and family [32]. Traditional beliefs, such as the perceived need for bowel rest, commonly influenced how ERAS nutrition recommendations were interpreted and followed. In another qualitative study looking at recommencing feeding after colorectal surgery, patients were often not given explanations regarding diet upgrades or downgrades after surgery, leading to fear and misunderstanding [33]. Patients described relying on their own interpretations of dietary changes rather than understanding diet progression as a structured component of recovery [33]. The post-discharge period emerged as a critical transition point where nutrition advice and professional support declined. When available, dietitians were consistently identified as trusted sources of perioperative nutrition information. However, access to dietetic input remained limited, particularly following discharge [34,35].

Physiological symptoms played a key role in shaping patients’ experiences of nutrition. Nausea, fatigue, pain, and altered bowel function were frequently reported as barriers to oral intake. Although ERAS protocols promote early feeding to support gastrointestinal recovery, patients often adjust their intake in response to symptom burden, interpreting postoperative symptoms as signals to reduce or withhold food. These behaviours were not explicitly linked to measured ERAS adherence, but instead appear to reflect patient-led self-regulation rather than non-compliance, consistent with ‘common sense’ coping responses [36]. When patients were given the opportunity to be involved in an individualised symptom management plan, they felt more empowered to engage with nutrition strategies within ERAS protocols [33].

The hospital environment and foodservice system also emerged as important determinants of patients’ abilities to engage with perioperative nutrition. Patients mostly described hospital food as unappealing, poorly tailored to individual needs, and at times inconsistent with nutrition messaging [37,38]. This affected their motivation to eat despite understanding its importance for recovery [39]. These findings suggest that the hospital food system is an active component of the nutritional care pathway, capable of either facilitating or hindering adherence to ERAS nutrition principles. These results highlight the potential to consider foodservice design and the broader eating environment as integral components of perioperative nutrition care.

Findings from this review indicate that clear, dietitian-led nutrition guidance embedded within perioperative ERAS pathways is essential to optimising patient engagement and the effectiveness of ERAS protocols. Patients consistently valued clear, nutrition-specific information across the peri- and postoperative journey. Recent systematic reviews from other surgical populations demonstrate associations between dietitian-led education and improved clinical and patient-reported outcomes. These include reduced length of stay, lower costs, and improved postoperative recovery outcomes [40,41]. Another recent systematic review looking at malnutrition in surgical patients identified that malnutrition has been repeatedly linked to adverse surgical outcomes, reinforcing the importance of early dietitian involvement and targeted nutrition counselling [42]. Individualised education appears particularly effective in supporting patient understanding and engagement, although group-based approaches also provide benefit in perioperative outcomes [40]. The 2017 ESPEN Clinical Surgery Guideline endorse postoperative dietary counselling for patients receiving perioperative nutrition therapy [43]. Together, these findings support the integration of dietitian input as a core component of ERAS care. Consistent nutrition education delivered in the perioperative and post-discharge period may help address information gaps at key transition points. This supports self-management and improves confidence in recovery [44,45]. Together, these strategies would position nutrition as a broad component of ERAS care with multiple touch points, supporting adherence, confidence and recovery across the perioperative journey.

Engaging patients in the service-level design and review of nutrition education may help bridge the gap between ERAS recommendations and lived recovery experiences. A codesign approach can be conceptualised as a quality improvement process. Patients would be involved as partners in refining how nutrition education is structured, delivered, and supported across an ERAS pathway. A codesign process was used in one of the studies by Gillis et al., which identified practical concerns around diet progression, symptom management, and uncertainty about recovery that were not apparent to clinicians alone [22]. Codesign approaches have similarly been shown to produce acceptable and effective nutrition education resources in an oncology population [46]. At a systems level, this approach may support the development of education that is more targeted, context-specific, and aligned with patient priorities. Decisions can be made about what information is needed, when it is best delivered (such as pre-operatively, inpatient recovery, post-discharge transition), and the most appropriate formats for delivery (such as written materials, standardised verbal messaging, digital resources, or structured face-to-face clinician follow-up). By moving beyond standardised, clinician-led information provision, codesigning education at the pathway or service level has the potential to improve relevance, acceptability, and patient confidence. This may enhance engagement and adherence to postoperative nutrition recommendations. Future research should evaluate the impact of such codesign-informed quality improvement initiatives on patient experience, satisfaction, and engagement with perioperative nutrition care.

A strength of this review was the novel focus on qualitative findings, including different methodological approaches and study designs for comprehensiveness. A limitation of the findings was the heterogeneity of the articles with respect to results and ERAS contexts. Nutrition was a primary focus in some studies but only an incidental finding in others, resulting in uneven depth and quality of nutrition-specific data. In addition, none of the studies provided consistent or detailed descriptions of ERAS protocol implementation, making it difficult to determine which components of ERAS were delivered and how comprehensively they were applied. This variability likely influenced patient experiences.

The confidence in the themes identified by this review should be interpreted in the light of the methodological quality of the included studies. The mixed-methods studies generally demonstrated lower methodological rigour, particularly regarding the integration of qualitative and quantitative components and the rationale for adopting a mixed-methods design. Several studies also relied primarily on survey-based designs, which are valuable for capturing broad patient perspectives but provide limited depth and contextual nuance compared with qualitative research. Consequently, some themes may be disproportionately influenced by studies with limited methodological transparency or by findings that are more descriptive than interpretive. The robustness and generalisability of the synthesised themes should be considered alongside these limitations.

This review also reflects patient perspectives exclusively. Although caregivers’ perspectives were eligible for inclusion, no caregiver-focused studies were identified. This represents an evidence gap given the potential role of caregivers in supporting nutrition-related recovery and adherence to ERAS recommendations. The absence of caregiver experiences may reflect the broader focus of ERAS research on patient experience and protocol adherence, with relatively little attention to the social and home-based context of postoperative recovery. Caregiver roles may be particularly influential after discharge, when responsibility for postoperative recovery shifts more heavily to the home environment. Notably, the discharge phase remains relatively underexplored compared with inpatient recovery in the ERAS literature.

Furthermore, the studies in this review were predominantly conducted in high-income Western countries, particularly in Europe, the United Kingdom and Canada. Healthcare infrastructure, cultural attitudes towards nutrition, and access to perioperative support may differ significantly in lower-resourced or culturally diverse settings. As a result, the experiences and barriers described by patients in these studies may not fully reflect those encountered in non-Western populations or in healthcare systems with different resource constraints. For example, challenges related to access to specialised nutrition support or differently structured perioperative care pathways may be more pronounced in low and middle-income countries. Cultural beliefs and practices around food, recovery, and the role of family or caregivers may also influence engagement with ERAS nutrition protocols. Therefore, while the themes identified in this review provide valuable insights into patient experiences within ERAS pathways, these contextual and cultural considerations should be taken into account when applying these findings to different healthcare settings. Future research should include more diverse populations and healthcare systems to enhance the global relevance of evidence on perioperative nutrition care.

While no language limits were applied to the literature searches conducted, the reviewers were only able to read English-language or English-translated papers. The search also did not include grey literature.

Publication bias should also be considered when interpreting these findings. Studies highlighting unmet needs or dissatisfaction with perioperative nutrition care may be more likely to be published than studies describing neutral or positive experiences. In qualitative research, self-selection may further shape the evidence base, as patients with stronger or more challenging experiences may be more inclined to participate in interviews, focus groups, or feedback-based studies. As a result, negative or problematic experiences may be more visible in the literature than the broader range of routine or satisfactory patient experiences within ERAS pathways.

## 5. Conclusions

In conclusion, this systematic review highlights the importance of positive patient engagement and education within colorectal ERAS protocols. While ERAS is a multidisciplinary model delivered as a bundle of components, this review shows that the nutrition elements of ERAS require specific attention to address patient barriers and expectations. In clinical practice, this could include embedding a standardised dietitian-led education pathway before surgery, with consistent messaging about nutrition recommendations, symptom expectations, and the purpose of postoperative diets and ONS. Clearly communicated recommendations can be made outlining expected diet progression and who to contact regarding nutrition concerns. Redesigning foodservice processes can also be considered to support recovery through more flexible meal access and providing food that is clinically appropriate and acceptable to patients. Embedding these approaches as part of ERAS protocols may improve patient engagement and nutritional outcomes.

## Figures and Tables

**Table 1:** Eligibility Criteria for a systematic review of patients’ perspectives on nutrition in Colorectal ERAS.

	Inclusion Criteria
Population	Any patients and caregivers of any age at any point in the perioperative period, including before surgery, immediately after surgery and long-term (more than six weeks post-surgery). Any patients undergoing colorectal surgery.
Intervention	Colorectal surgery utilising the ERAS pathway.
Comparator	No comparator.
Outcome	Perceptions, experiences, attitudes, knowledge or views towards the nutrition component of ERAS reported by patients or caregivers. Studies were eligible if they reported patient experiences of nutrition either as a primary focus or as a clearly identifiable component of a broader exploration of ERAS care. Broader studies were retained when they provided extractable data relevant to perioperative nutrition, because patient experiences of nutrition in ERAS are often embedded within wider findings of recovery, symptom management, and discharge care rather than reported as primary or standalone outcomes. This may include: - Studies that explore what patients were thinking about their nutrition during the perioperative period. - Studies exploring the level of patient knowledge about the importance of nutrition in their surgical recovery. - Patients’ attitudes toward nutrition guidelines or protocols provided as part of ERAS. - Descriptions of patient experiences with nutrition care and support during the perioperative period. Includes interactions with dietitians, different routes of nutritional support they received, and successes or challenges they faced in these interactions or with these nutritional support routes.
Study Design	Primary research using qualitative, quantitative or mixed methods design to collect descriptive data through surveys, focus groups, interviews or observation.

**Table 2:** Characteristics of included studies.

Study	Study Aim(s)	Language	Country	Sample Size	Study Design
Brundrett et al. 2011 [17]	To establish if patients were being offered high-energy, high-protein drinks postoperatively as recommended by their enhanced recovery pathway and to obtain patients’ views on these supplements.	English	United Kingdom	17 patients	Quantitative Descriptive Survey
Gillis et al. 2017 [22]	Explore the experience of patients undergoing colorectal surgery within an Enhanced Recovery After Surgery (ERAS) programme. Use these experiential data to inform the development of a framework to support ongoing, meaningful patient engagement in ERAS.	English	Canada	17 patients	Qualitative
Gillis et al. 2019 [23]	A nutrition-focused qualitative analysis of patient experience and of ERAS implementation across our province was conducted to better understand the barriers to successful adoption of ERAS nutrition elements.	English	Canada	27 patients	Qualitative
Samuelsson et al. 2018 [25]	To describe how older patients experience the healthcare chain and information given before, during and after colorectal cancer surgery.	Study conducted in Swedish. Paper written in English.	Sweden	16 patients	Qualitative
Partoune et al. 2017 [24]	To evaluate the feelings and satisfaction of patients when at home after an ERP for colorectal surgery.	Study conducted in French. Paper written in English.	Belgium	41 patients	Quantitative Descriptive Survey
Cooper 2013 [21]	To measure the experience of patients cared for on a colorectal ER pathway.	English	United Kingdom	48 patients	Mixed methods
Burch 2015 [18]	To review patients’ experiences of a colorectal enhanced recovery pathway to enable service evaluation and improvement.	English	United Kingdom	54 patients	Mixed methods
Taylor and Burch 2011 [27]	To examine service users’ views on an enhanced recovery programme (ERP) for colorectal surgery patients, to improve service provision.	English	United Kingdom	10 patients	Qualitative
Wennstrom et al. 2020 [28]	To describe patient experiences of health within the concept of ERAS after colorectal surgery during a hospital stay and within 2 weeks of discharge.	Study conducted in Swedish. Paper written in English.	Sweden	80 patients	Mixed Methods
Short et al. 2015 [26]	To explore the perioperative nutrition experiences of colorectal surgical patients to identify barriers and facilitators to the integration of nutrition within ERAS.	English	United Kingdom	16 patients	Qualitative
Olivares et al. 2018 [20]	To determine the satisfaction of patients following the implementation of an ERAS protocol in elective colorectal surgery.	English	Spain	55 patients	Quantitative Descriptive Survey
Burch and Taylor 2012 [19]	To identify how well patients recover in the period between discharge home from the hospital and surgical follow-up in The outpatient clinic will discuss their support needs following participation in the ERAS programme.	English	United Kingdom	100 patients	Mixed Methods
Aasa et al. 2013 [15]	To identify and describe patients’ experiences of a preoperative information session with a nurse, as part of the enhanced recovery after surgery (ERAS) concept, and its impact on patient participation in their own care.	English	Sweden	12 patients	Qualitative
Xu et al. 2024 [29]	To explore the knowledge, attitude, and practice (KAP) towards enhanced recovery after surgery (ERAS) among colorectal cancer (CRC) patients.	English	China	652 patients	Quantitative descriptive cross-sectional survey
S Ben Ali et al. 2024 [16]	To assess the extent to which patient activation (PA, i.e., knowledge, skills, and confidence to participate in care) is associated with adherence to an ER pathway for colorectal surgery.	English	Canada	286 patients	Quantitative Survey

**Table 3:** Quality assessment of included studies using the Mixed Methods Appraisal Tool (MMAT) version 2018 [13].

Article	Study Design	MMAT Criteria Applied	Q1	Q2	Q3	Q4	Q5
Brundrett et al. et al. 2011 [17]	Quantitative Descriptive	QD1–QD5	?	?	?	Y	Y
Gillis 2017 et al. [22]	Qualitative	QL1–QL5	Y	Y	Y	Y	Y
Gillis 2019 et al. [23]	Qualitative	QL1–QL5	Y	Y	Y	Y	Y
Samuelsson et al. 2018 [25]	Qualitative	QL1–QL5	Y	Y	Y	Y	Y
Partoune et al. 2017 [24]	Quantitative Descriptive	QD1–QD5	Y	Y	Y	Y	Y
Cooper 2013 [21]	Mixed methods	MM1–MM5	Y	Y	Y	Y	N
Burch 2015 [18]	Mixed methods	MM1–MM5	Y	Y	Y	Y	Y
Taylor and Burch 2011 [27]	Qualitative	QL1–QL5	Y	Y	Y	Y	Y
Burch and Taylor 2012 [19]	Mixed methods	MM1–MM5	N	N	N	N	N
Wennstrom et al. 2020 [28]	Mixed methods	MM1–MM5	Y	Y	Y	?	Y
Short et al. 2015 [26]	Qualitative	QL1–QL5	Y	Y	Y	Y	Y
Olivares et al. 2018 [20]	Quantitative Descriptive	QD1–QD5	Y	Y	Y	Y	Y
Aasa et al. 2013 [15]	Qualitative	QL1–QL5	Y	Y	Y	Y	Y
Xu 2024 [29]	Quantitative Descriptive	QD1–QD5	Y	Y	Y	Y	Y
S Ben Ali et al. 2024 [16]	Quantitative Descriptive	QD1–QD5	Y	Y	Y	Y	Y

Abbreviations: Y = Yes; N = No; ? = unable to tell. Qualitative criteria (QL1–QL5): QL1: Appropriateness of the qualitative approach to the research question; QL2: Adequacy of qualitative data collection methods; QL3: Derivation of findings from the data; QL4: Substantiation of interpretations by the data; QL5: Coherence between data sources, collection, analysis, and interpretation. Quantitative descriptive criteria (QD1–QD5): QD1: Relevance of the sampling strategy to the research question; QD2: Representativeness of the sample to the target population; QD3: Appropriateness of measurements; QD4: Risk of nonresponse bias; QD5: Appropriateness of statistical analysis. Mixed-methods criteria (MM1–MM5): MM1: Rationale for using a mixed-methods design; MM2: Integration of qualitative and quantitative components; MM3: Interpretation of integrated outputs; MM4: Management of divergences or inconsistencies between qualitative and quantitative results; MM5: Adherence of each component to its respective quality criteria.

## Data Availability

The data that support the findings of this study are available within the article and its Supplementary Material. Data sharing is not applicable to this article as no new datasets were created or analysed in this study.

## Supplementary Materials

The following supporting information can be downloaded at: https://www.mdpi.com/article/10.3390/nu18111790/s1, Table S1: PRISMA-2020-Checklist; Table S2: Search Terms for Ovid MEDLine, Embase and Emcare; Table S3: Search Terms for CINAHL; Table S4: Search Strings for OVID MEDLine, Embase and Emcare; Table S5: Mapping of included studies to themes and key findings; File S1: Search Strings for CINAHL.
