# Supplementary material for: Patient Experiences of Nutrition in Enhanced Recovery After Colorectal Surgery: A Systematic Review
Source: Nutrients. 2026 Jun 1;18(11):1790. doi: 10.3390/nu18111790 (PMC13258613; doi:10.3390/nu18111790)
Supplement: Supplementary file 1 [file nutrients-18-01790-s001.zip › nutrients-4320805-supplementary.pdf]

Supplementary Material

Table 1: Search Terms for Ovid MEDLine, Embase and Emcare

Key Concepts	ERAS<br>*Field 1*	Patient experience<br>*Field 2*	Colorectal surgery<br>*Field 3*
Related terms	1. Enhanced Recovery After Surgery<br>2. enhanced adj2 recovery<br>3. eras - search as keyword<br>4. (Perioperat* OR peri operat*) adj3 (protocol* or pathway* or bundle* or program* or nutriti*)<br>5. (postoperat* or post operat*) adj3 (protocol* or pathway* or bundle* or program* or nutriti*)	1. patient attitude* or patient engagement* or patient preference* or patient satisfaction*<br>2. (Patient* or consumer* or end user*) adj3 (experience* or attitude* or satisfact* or percept* or engag* or knowledg* or understand* or reflect* or view* or opinion* or prefer*)	1. colorectal surgery - Map to Subject heading – check 'explode' combine with OR<br>2. Colon – explode – subheading surgery<br>3. Rectum –explode- subheading surgery<br>4. (colorectal or colon or rectal or lower gastrointestin* or lower GI or abdo*) adj3 (surg* or operat* or resect*)<br>Combine with OR

	6. (preoperat* or pre operat*) adj3 (protocol* or pathway* or bundle* or program* or nutriti*)<br>7. (pre habilitat* or prehabilitat) adj3 (protocol* or pathway * or bundle* or program* or nutriti*)	Combine with OR	

Combine all 3 fields with AND

Table 2: Search Terms for CINAHL

Key Concepts	ERAS<br>*Field 1*	Patient experience<br>*Field 2*	Colorectal surgery<br>*Field 3*
Related terms	1. Enhanced Recovery After Surgery - find all my search terms	Find any of my search terms	Find any of my search terms

	2. enhanced N2 recovery<br>3. eras - search as keyword<br>4. (Perioperat* OR peri operat*) N3 (protocol* or pathway* or bundle* or program* or nutriti*)<br>5. (postoperat* or post operat*) N3 (protocol* or pathway* or bundle* or program* or nutriti*)<br>6. (preoperat* or pre operat*) N3 (protocol* or pathway* or bundle* or program* or nutriti*)	1. patient attitude* or patient engagement* or patient preference* or patient satisfact*<br>2. (Patient* or consumer* or end user*) N3 (experience* or attitude* or satisfact* or percept* or engag* or knowledg* or understand* or reflect* or view* or opinion* or prefer*)	1. colorectal surgery - apply related words, search within full text, apply equivalent subjects<br>2. Colon – apply related words, search within full text, apply equivalent subjects<br>3. Rectum - apply related words, search within full text, apply equivalent subjects<br>4. (colorectal or colon or rectal or lower gastrointestin* or lower GI* or abdo*) N3 (surg* or resect* or operat*)<br><br>Combine with OR

	7. (pre habilitat* or prehabilitat) N3 (protocol* or pathway * or bundle* or program* or nutriti*)<br><br>Combine with OR		

Combine all 3 fields with AND

Table 3: Search Strings for OVID MEDLine, Embase and Emcare

#	**Query**
1	exp colorectal surgery/
2	exp colon/su [Surgery]
3	exp rectum/su [Surgery]
4	((colorectal or colon or rectal) adj3 (surg* or operat* or resect*)).mp. [mp=title, book title, abstract, original title, name of substance word, subject heading word, floating sub-heading word, keyword heading word, organism supplementary concept word, protocol supplementary concept word, rare disease supplementary concept word, unique identifier, synonyms, population supplementary concept word, anatomy supplementary concept word]
5	1 or 2 or 3 or 4
6	enhanced recovery after surgery/

7	(enhanced adj2 recovery).mp. [mp=title, book title, abstract, original title, name of substance word, subject heading word, floating sub-heading word, keyword heading word, organism supplementary concept word, protocol supplementary concept word, rare disease supplementary concept word, unique identifier, synonyms, population supplementary concept word, anatomy supplementary concept word]
8	eras.mp. [mp=title, book title, abstract, original title, name of substance word, subject heading word, floating sub-heading word, keyword heading word, organism supplementary concept word, protocol supplementary concept word, rare disease supplementary concept word, unique identifier, synonyms, population supplementary concept word, anatomy supplementary concept word]
9	((perioperat* or peri operat*) adj3 (protocol* or pathway* or bundle*)).mp. [mp=title, book title, abstract, original title, name of substance word, subject heading word, floating sub-heading word, keyword heading word, organism supplementary concept word, protocol supplementary concept word, rare disease supplementary concept word, unique identifier, synonyms, population supplementary concept word, anatomy supplementary concept word]
10	((postoperat* or post operat*) adj3 (protocol* or pathway* or bundle*)).mp. [mp=title, book title, abstract, original title, name of substance word, subject heading word, floating sub-heading word, keyword heading word, organism supplementary concept word, protocol supplementary concept word, rare disease supplementary concept word, unique identifier, synonyms, population supplementary concept word, anatomy supplementary concept word]
11	((pre habilit* or prehabilit*) adj3 (protocol* or pathway* or bundle* or program*)).mp. [mp=title, book title, abstract, original title, name of substance word, subject heading word, floating sub-heading word, keyword heading word, organism supplementary concept word, protocol supplementary concept word, rare disease supplementary concept word, unique identifier, synonyms, population supplementary concept word, anatomy supplementary concept word]
12	6 or 7 or 8 or 9 or 10 or 11

13	5 and 12
14	patient attitude/ or patient engagement/ or patient preference/ or patient satisfaction/
15	((patient* or consumer* or end user*) adj3 (experience* or attitude* or satisfact* or perspect* or percept* or engag* or knowledg*)).mp. [mp=title, book title, abstract, original title, name of substance word, subject heading word, floating sub-heading word, keyword heading word, organism supplementary concept word, protocol supplementary concept word, rare disease supplementary concept word, unique identifier, synonyms, population supplementary concept word, anatomy supplementary concept word]
16	14 or 15
17	13 and 16
18	limit 17 to english language

Appendix 1: Search Strings for CINAHL

EBSCO Export

S#: S18

Combined search

Query (user-entered): S16 AND S17

Query (expanded/display term): (((colorectal surgery) OR (colon) OR (rectum) OR ((colorectal or colon or rectal or lower gastrointestin* or lower GI* or abdo*) N3 (surg* or resect* or operat*))) AND ((enhanced recovery after surgery) OR (enhanced N2 recovery) OR (eras) OR ((Perioperat* OR peri operat*) N3 (protocol* or pathway* or bundle* or program* or nutriti*)) OR ((postoperat* or post operat*) N3 (protocol* or pathway* or bundle* or program* or nutriti*)) OR ((preoperat* or pre operat*) N3 (protocol* or pathway* or bundle* or program* or nutriti*)) OR ((pre habilitat* or prehabilitat) N3 (protocol* or pathway * or bundle* or program* or nutriti*)))) AND ((patient attitude* or patient engagement* or patient preference* or patient satisfact*) OR ((Patient* or consumer* or end user*) N3 (experience* or attitude* or satisfact* or percept* or engag* or knowledg* or understand* or reflect* or view* or opinion* or prefer*)))

Search run Date (ISO): 2026-05-23T03:09:44.216Z

Results (count): 9017

Search Mode: Proximity

Number of databases: 1

Database(s): CINAHL Complete

Interface: EBSCOhost Research Databases

Table 4: Mapping of included studies to themes and key findings

**Study**	**Themes present**	**Relevant findings**
Brundrett et al. 2011 [17]	3	Theme 3: Some patients found ONS easy to take, but others struggled with the texture, sweetness, or heaviness of the drinks. Problems included the artificial taste and difficulty finishing the drinks.
Gillis et al. 2017 [22]	1, 2,4, 5	Theme 1: Patients who understood the reasoning for ERAS components were more likely to follow them.<br><br>Theme 2: Patients wanted clearer guidance on suitable foods relatives could bring from home, and noted the lack of food availability outside scheduled mealtimes.<br><br>Theme 4: Patients perceived the lack of nutrition information as a missed opportunity.<br><br>Patients reported rapport with the nursing staff was variable, with some nurses being very informative and some

		being less interested. Some nurses appeared to be fixated on following the ERAS protocol regardless of patients' individual conditions.<br><br>Theme 5: patients reported confusion about postoperative diet expectations at home as they did not feel they received adequate information for self-management.
Gillis et al. 2019 [23]	1,2, 4	Theme 1: Many patients believed in the healing value of nutritious foods, a view reinforced by the nutrition education principles of ERAS. A misconception was the bowel needed "rest" before food could be safely resumed, delaying oral intake despite ERAS advocating for early feeding. Some misinterpreted carbohydrate loading as "more is better" or viewed low-fibre transition diets as unhealthy, leading to poor adherence.<br><br>Theme 2: Complaints about hospital food were common, with dissatisfaction stemming from both misunderstanding the purpose of the postoperative transition diet and personal taste preferences.<br><br>Theme 4: Some patients distrusted providers or the food system when messages conflicted with their own beliefs or between staff members.

		Contradictory advice on early feeding and the transition diet caused confusion. Some providers told patients they had no dietary restrictions, which contradicted with the low-fibre post-surgical transition diet implemented by the healthcare system. Staff also sometimes criticised the food they encouraged patients to eat.
Samulesson et al. 2018 [25]	2, 4, 5	Theme 2: Many felt nutrition advice was too generic, with no tailoring to individual symptoms such as nausea, fatigue, or pain.<br>Participants were surprised that something as vital to recovery as adequate, nourishing food was neglected, expressing frustration at hospital food quality.<br><br>Theme 4: medical staff were perceived as busy or under stress and communication was perceived as hasty. This caused the feelings of a loss of control and anxiety, hindering patient engagement in their recovery.<br><br>Theme 5: patients had many questions in the postoperative period and were unsure who to contact. Few received dietitian follow-up at home. When they did, it was highly valued for reassurance and monitoring of their nutrition status.
Partoune et al. 2017 [24]	2	Theme 2: Most were satisfied with early diet resumption, though about a third struggled with eating normally at home

		due to appetite loss or digestive discomfort. A few believed a slower diet progression would have helped.
Cooper 2013 [21]	2	Theme 2: Food was one of the most common criticisms of hospital stay, with requests for better-quality meals.
Burch 2015 [18]	2, 4	Theme 2: Food was one of the most common criticisms of hospital stay, with requests for better-quality meals.<br><br>Theme 4: Nursing and multidisciplinary care received good feedback, though food quality and staffing shortages were ongoing concerns.
Taylor and Burch 2011 [27]	1, 2, 3	Theme 1: Food was an importance emotional component of Enhanced Recovery Programs to patients.<br><br>Theme 2: unappetising hospital food was a factor hindering engagement with eating.<br><br>Theme 3: ONS drinks were viewed as tolerable however not always pleasant.
Wennstrom et al. 2020 [28]	1, 2, 5	Theme 1: Information was perceived as inadequate and sometimes incorrect. Patients believed there was a risk to early oral feeding after surgery due to slowed-down bowel movements.

		Theme 2: Poor appetite was common, with over half reporting reduced or no appetite. Food sometimes served as a social connector, with patients enjoying dining room interactions with other patients.<br><br>Theme 5: all patients appreciated the follow up call made by a nurse after discharge as it gave them the feeling of safety.
Short et al. 2015 [26]	1, 2, 3, 4, 5	Theme 1: Adjusting to a stoma made food feel burdensome for some. Uncertainty about safe food choices led some to intentionally reducing eating to limit stoma output.<br><br>Theme 2: Many patients were aware of the recommendation for early resumption of diet and were pleased to accept responsibility for this component of ERAS. However, nausea, vomiting, or fear of these symptoms reduced willingness to eat. Some accepted lower appetite in hospital as normal due to reduced activity levels compared to when at home. Patients generally appreciated food variety but worried that choice might come at the cost of quality.<br><br>Theme 3: Most patients knew when to take preoperative drinks but were unclear on the difference between carbohydrate loading and nutritional supplements. Drinks were unappealing but seen as beneficial to prepare for surgery. There was less willingness to consume them after surgery.

		Theme 4: Staff were seen as approachable and supportive, giving patients confidence to request food and advice.<br><br>Theme 5: Going home was expected to help restore appetite and normalise eating patterns, though some worried about adapting to lifestyle changes and establishing a new routine.
Olivares et al. 2018 [20]	1	Theme 1: Opinions on the timing of oral fluid introduction postoperatively varied, with most patients feeling it was too soon to have oral fluids within 24 hours post-surgery.
Burch and Taylor 2012 [19]	5	Theme 5: Concerns raised post-discharge were dietary intake, fatigue and bowel function issues. Patients were concerned about eating half or less of their normal amounts of food after returning home.
Aasa et al. 2013 [15]	4	Theme 4: Patients appreciated involvement in tracking their food intake but felt that hospital staff failed to reinforce or follow up on preoperative advice during the stay, leaving uncertainty about responsibility for ensuring dietary compliance.
Xu et al. 2024 [29]	1, 4	Theme 1: Greater ERAS knowledge was linked with better attitudes and practices, though factors like age, BMI, and profession also influenced adherence.<br><br>Theme 4: 75.07% of patients felt receiving nutritional support before surgery would be helpful for them [28].

S Ben Ali et al. 2024 [16]	1	Theme 1: Higher patient activation by combining confidence, knowledge, and self-management skills was strongly associated with adherence to nutrition-related ERAS components of early oral intake and ONS use.

Table 5 PRISMA-2020-Checklist [11]

Section and Topic	Item #	Checklist item	Location where item is reported
**TITLE**			
Title	1	Identify the report as a systematic review.	Page 1
**ABSTRACT**			
Abstract	2	See the PRISMA 2020 for Abstracts checklist.	Page 1
**INTRODUCTION**			
Rationale	3	Describe the rationale for the review in the context of existing knowledge.	Page 2-3
Objectives	4	Provide an explicit statement of the objective(s) or question(s) the review addresses.	Page 3
**METHODS**			
Eligibility criteria	5	Specify the inclusion and exclusion criteria for the review and how studies were grouped for the syntheses.	Page 3
Information sources	6	Specify all databases, registers, websites, organisations, reference lists and other sources searched or consulted to identify studies. Specify the date when each source was last searched or consulted.	Page 4
Search strategy	7	Present the full search strategies for all databases, registers and websites, including any filters and limits used.	Page 4
Selection process	8	Specify the methods used to decide whether a study met the inclusion criteria of the review, including how many reviewers screened each record and each report retrieved, whether they worked independently, and if applicable, details of automation tools used in the process.	Page 4
Data collection process	9	Specify the methods used to collect data from reports, including how many reviewers collected data from each report, whether they worked independently, any processes for obtaining or confirming data from study investigators, and if applicable, details of automation tools used in the process.	Page 4
Data items	10a	List and define all outcomes for which data were sought. Specify whether all results that were compatible with each outcome domain in each study were sought (e.g. for all measures, time points, analyses), and if not, the methods used to decide which results to collect.	Page 4-5
	10b	List and define all other variables for which data were sought (e.g. participant and intervention characteristics, funding sources). Describe any assumptions made about any missing or unclear information.	Page 4-5
Study risk of bias assessment	11	Specify the methods used to assess risk of bias in the included studies, including details of the tool(s) used, how many reviewers assessed each study and whether they worked independently, and if applicable, details of automation tools used in the process.	Page 5
Effect measures	12	Specify for each outcome the effect measure(s) (e.g. risk ratio, mean difference) used in the synthesis or presentation of results.	N/A
Synthesis methods	13a	Describe the processes used to decide which studies were eligible for each synthesis (e.g. tabulating the study intervention characteristics and comparing against the planned groups for each synthesis (item #5)).	Page 5
	13b	Describe any methods required to prepare the data for presentation or synthesis, such as handling of missing summary statistics, or data conversions.	Page 5
	13c	Describe any methods used to tabulate or visually display results of individual studies and syntheses.	Page 5

Section and Topic	Item #	Checklist item	Location where item is reported
	13d	Describe any methods used to synthesize results and provide a rationale for the choice(s). If meta-analysis was performed, describe the model(s), method(s) to identify the presence and extent of statistical heterogeneity, and software package(s) used.	Page 5
	13e	Describe any methods used to explore possible causes of heterogeneity among study results (e.g. subgroup analysis, meta-regression).	Page 5
	13f	Describe any sensitivity analyses conducted to assess robustness of the synthesized results.	Page 5
Reporting bias assessment	14	Describe any methods used to assess risk of bias due to missing results in a synthesis (arising from reporting biases).	Page 4-5
Certainty assessment	15	Describe any methods used to assess certainty (or confidence) in the body of evidence for an outcome.	Page 5
**RESULTS**			
Study selection	16a	Describe the results of the search and selection process, from the number of records identified in the search to the number of studies included in the review, ideally using a flow diagram.	Page 5
	16b	Cite studies that might appear to meet the inclusion criteria, but which were excluded, and explain why they were excluded.	Page 6
Study characteristics	17	Cite each included study and present its characteristics.	Page 6-8
Risk of bias in studies	18	Present assessments of risk of bias for each included study.	Page 8-9
Results of individual studies	19	For all outcomes, present, for each study: (a) summary statistics for each group (where appropriate) and (b) an effect estimate and its precision (e.g. confidence/credible interval), ideally using structured tables or plots.	Page 6-8
Results of syntheses	20a	For each synthesis, briefly summarise the characteristics and risk of bias among contributing studies.	Page 8
	20b	Present results of all statistical syntheses conducted. If meta-analysis was done, present for each the summary estimate and its precision (e.g. confidence/credible interval) and measures of statistical heterogeneity. If comparing groups, describe the direction of the effect.	Page 9-15
	20c	Present results of all investigations of possible causes of heterogeneity among study results.	Page 9-15
	20d	Present results of all sensitivity analyses conducted to assess the robustness of the synthesized results.	Page 9-15
Reporting biases	21	Present assessments of risk of bias due to missing results (arising from reporting biases) for each synthesis assessed.	N/A
Certainty of evidence	22	Present assessments of certainty (or confidence) in the body of evidence for each outcome assessed.	N/A
**DISCUSSION**			
Discussion	23a	Provide a general interpretation of the results in the context of other evidence.	Page 15-18
	23b	Discuss any limitations of the evidence included in the review.	Page 17-18
	23c	Discuss any limitations of the review processes used.	Page 17-18
	23d	Discuss implications of the results for practice, policy, and future research.	Page 17-18
**OTHER INFORMATION**			
Registration and protocol	24a	Provide registration information for the review, including register name and registration number, or state that the review was not registered.	Page 3
	24b	Indicate where the review protocol can be accessed, or state that a protocol was not prepared.	Page 3
	24c	Describe and explain any amendments to information provided at registration or in the	Page 3

Section and Topic	Item #	Checklist item	Location where item is reported
		protocol.	
Support	25	Describe sources of financial or non-financial support for the review, and the role of the funders or sponsors in the review.	Page 18
Competing interests	26	Declare any competing interests of review authors.	Page 18
Availability of data, code and other materials	27	Report which of the following are publicly available and where they can be found: template data collection forms; data extracted from included studies; data used for all analyses; analytic code; any other materials used in the review.	Page 18
